# Eosinophilic gastrointestinal disorders: variations in eosinophilic counts among investigators and staining methods

**DOI:** 10.20407/fmj.2024-019

**Published:** 2024-12-27

**Authors:** Mina Ikeda, Satoshi Arakawa, Takashi Kobayashi, Ken-ichi Inada, Yuka Kiriyama, Takahiko Sakuma, Takuma Ishihara, Akiko Yagami, Kayoko Suzuki, Kyoko Futamura, Senju Hashimoto, Hironao Miyoshi, Satoshi Yamamoto, Haruhiko Tachino, Yoshihiro Imaeda, Hiroyuki Kato, Yukio Asano, Yoshiaki Katano, Akihiko Horiguchi

**Affiliations:** 1 Department of Gastroenterology, Fujita Health University Bantane Hospital, Nagoya, Aichi, Japan; 2 Department of Gastroenterological Surgery, Fujita Health University Bantane Hospital, Nagoya, Aichi, Japan; 3 Fujita Health University General Allergy Center, Nagoya, Aichi, Japan; 4 Department of Diagnostic Pathology, Fujita Health University Hospital, Toyoake, Aichi, Japan; 5 Department of Diagnostic Pathology, Toyohashi Medical Center, Toyohashi, Aichi, Japan; 6 Innovative and Clinical Research Promotion Center, Gifu University Hospital, Gifu, Gifu, Japan; 7 Department of Allergology, Fujita Health University, School of Medicine, Nagoya, Aichi, Japan; 8 Medical Corporation Yuukikai, Health Care Facilities for the Elderly requiring Long-term Care, Handa, Aichi, Japan; 9 Department of Clinical Laboratory, Holy Spirit Hospital, Nagoya, Aichi, Japan

**Keywords:** Commission International de l’Eclairage (CIE), Eosinophilic esophagitis, Eosinophilic gastroenteritis, Direct fast scarlet stain, Linear mixed-effect model

## Abstract

**Objective:**

Eosinophilic gastrointestinal disorders (EGIDs) are pathologically diagnosed by manually counting the eosinophils in biopsy tissue under microscopy. However, the skill of the individual examiner is considered to influence the accuracy of the resulting eosinophil count (EC). This study aimed to examine the effects of different examiners and histopathological staining types on the EC results of pathological tissues from patients with EGIDs.

**Methods:**

Infiltrating eosinophils in lesion tissues from 10 eosinophilic esophagitis and 28 eosinophilic gastroenteritis cases were counted by three pathologists and one cytotechnologist. The intra- and inter-observer variabilities in ECs related to hematoxylin-eosin (HE), May-Grünwald Giemsa (MG), and direct fast scarlet (DFS) staining were investigated. The effects of examiner expertise and staining method on ECs were analyzed using a linear mixed effects model. The difference in color value (ΔE) for each staining method was obtained using the Commission International de l’Eclairage luminance-a-b model (L*a*b*).

**Results:**

There was no significant intra-observer variability in eosinophil counting. Regarding inter-observer agreement, the examiner with the most EGIDs experience reported higher ECs than the other examiners for all three staining types (*P*<0.001). ECs were significantly higher with MG and DFS staining than with HE staining, regardless of the examiner (both *P*<0.001). Additionally, the ΔE values with DFS were higher than those with MG and HE.

**Conclusions:**

DFS staining offered the most selective visualization of eosinophils. ECs may vary depending on both the skill of the examiner and the staining method.

## Introduction

Eosinophilic gastrointestinal disorders (EGIDs) are marked by high infiltration of eosinophils into the gastrointestinal mucosa, causing chronic inflammation. Gastrointestinal dysfunction and other disease manifestations may appear in EGIDs, in addition to recurrence and relapse.^1,2^ Eosinophilic esophagitis (EoE) is a localized lesion in the esophagus, whereas eosinophilic gastroenteritis (EGE) lesions occur in the esophagus as well as the gastrointestinal tract, both resulting from eosinophil infiltration. EoE was first reported in 1978,^3^ and EGE in 1937.^4^ Several studies have reported that EGIDs are caused by food allergies and type 2 helper T cell (Th2) immunological responses, with sensitized antigens acting as allergens.^5–7^ In recent years, while the prevalence of EoE has increased by 50- to 70-fold in developed Western countries,^8^ the prevalence of EGE has not.^9^ The main diagnostic criteria for EGE are eosinophil infiltration in the gastrointestinal tract and abdominal symptoms originating from the gastrointestinal tract.^10^ The definitive diagnosis of EGE relies on gastrointestinal endoscopy and histopathology, particularly the determination of the total number of infiltrating eosinophils per high-power field (eos/HPF).^11–15^ EoE is diagnosed as the presence of ≥15 eos/HPF under hematoxylin-eosin (HE) staining of esophageal biopsies.^16^ The EGE diagnosis includes abdominal symptoms, such as pain and diarrhea, and the presence of a dense eosinophilic infiltrate through gastrointestinal biopsies.^17^ In Japan, EGE is defined as abdominal symptoms, infiltration of inflammatory cells mainly comprising eosinophils in the mucosa of the gastrointestinal tract, ascites and multiple eosinophils in ascites, history of allergic disease, high peripheral eosinophil counts (ECs), gastrointestinal wall thickening identified on computed tomography (CT), edema, redness, erosion of the gastrointestinal tract on endoscopy, and effective response to glucocorticoid therapy.^17,18^ However, the peak eosinophil density in the terminal ileum and cecum can occasionally reach ≥20 eos/HPF under HE staining, even in healthy individuals. Blood examinations have reportedly detected increases in the peripheral blood eosinophils of 30% in EoE and 80% in EGE.^19^ A blood examination is useful in the diagnosis of EGIDs; however, ECs are not necessarily elevated. The endoscopic findings of EoE include longitudinal furrows, multiple ring-shaped stenoses, and white plaques. In a study of EoE, at least one abnormality was detected by endoscopy in 93% of cases.^20^ Endoscopic findings of EGE include mucosal edema, redness, and erosion, but there are no specific findings.^19^ Therefore, histopathological examination is indispensable for EGIDs.

Histopathological diagnosis of EGIDs is based on HE staining, and thus eosinophils are likely to be counted using HE-stained slides. However, the staining method is not specified in guidelines. Eosinophils are approximately 17×12 μm in size, exhibit double-lobed nuclei, and contain approximately 200 cytoplasmic granules of 500–1000 nm in size.^21,22^ These granules appear bright pink under HE staining and may be mistaken for red blood cells, plasma cell cytoplasm, or background stromal collagen fiber fragments if the nucleus is outside the paraffin section, or the granules are scattered around the eosinophil during partial degranulation, or the collagen fibers are frayed and cut in a cross-sectional manner.

Eosinophils and other cellular components such as red blood cells, plasma cell cytoplasm, or stromal collagen fiber fragments may appear differently depending on the staining method used. Direct fast scarlet (DFS), like Congo red, is often used for amyloid staining; however, of the two, it is easier to adjust the procedures and reagents for DFS. Tanaka et al. reported that DFS staining is also useful for the diagnosis of EoE.^23^ May-Grünwald Giemsa (MG) is usually used for blood, bone marrow samples, and parasites, but is also reportedly useful for eosinophil staining.^24^ Therefore, we hypothesized that these two staining methods might contribute to the evaluation of ECs.

Human color perception varies from person to person, and the color temperature of the microscopic field of view depends on the power setting of the light source of the microscope. In an effort to standardize and numerically characterize the color features of eosinophils and other cell types and components of EoE and EGE specimens, photomicrographs were captured and digitally analyzed for brightness/contrast and color saturation (yellow/green and red/blue axes, respectively). Using the Commission International de l’Eclairage (CIE) model,^25^ we were able to objectively assess the differences in the color representation of eosinophil granules and other cell types and histological components.

Eosinophils stained with MG or DFS may be counted more accurately than HE, which could be useful for the diagnosis and treatment of EGIDs. While the current diagnostic criteria for EGE in the Japanese guidelines include an EC of ≥20/HPF, caution is required because even healthy individuals may show EC of ≥20/HPF in the right hemi-colon. Indeed, there have been many reports on this phenomenon.^26,27^ At our hospital, we have seen many colon biopsy samples containing ≥20 eos/HPF from patients suspected of an EGIDs. To confirm the diagnosis of such patients, we first seek an accurate EC, which may allow for the true cut-off value to be obtained. To our knowledge, this is the first study to examine differences in ECs of EGIDs biopsy tissues using HE, MG, and DFS staining methods. We investigated whether MG or DFS staining could yield more accurate ECs compared with HE, and also analyzed intra- and inter-observer variations in the ECs.

## Methods

Ten patients with EoE and 28 with EGE diagnosed in the General Allergy Center of Fujita Health University Bantane Hospital from January 2016 to June 2019 were included in this study. Patient background information is summarized in Table 1. The opt-out informed consent procedure and data analysis protocols were reviewed and approved by the Fujita Health University Certified Review Board (approval numbers CI20-103 and HM20-068, respectively). Patients with inflammatory bowel disease, parasitic infections, eosinophilic hyperplasia syndrome, Churg-Strauss syndrome, and malignancy were not included in this study. All cases were diagnosed as EoE or EGE by integrating medical history, clinical symptoms, laboratory data, endoscopic findings, and histological evaluation of tissue eosinophils in the endoscopic biopsy samples.

### Pathological examinations

**Figure 1:**
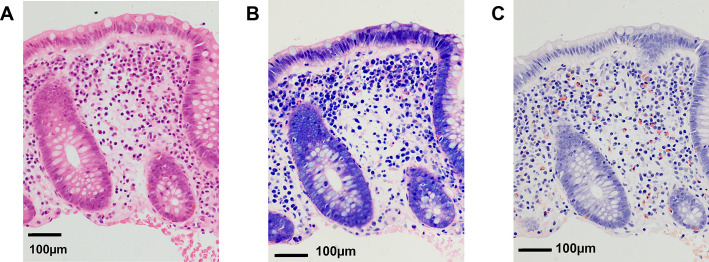
Appearance of eosinophils under different staining methods. **A** Hematoxylin - eosin (HE) stain. The eosinophil cytoplasm is conspicuously pink; however, the shade of pink is similar to that of plasma cell cytoplasm and erythrocytes. Thus, careful observation of nuclear features and cytoplasmic granules is required to distinguish eosinophils from plasma cells and erythrocytes. **B** May-Grünwald Giemsa stain. Note that eosinophils are bright pink, whereas plasma cells and erythrocytes are pale pink. Eosinophils are easier to detect with this stain than with HE stain. **C** Direct fast scarlet stain. Note that the eosinophil cytoplasm is selectively stained orange, making it easy to distinguish eosinophils from other cells, including erythrocytes. Scale bars=100 μm (**A**–**C**).

Biopsy specimens were taken from the middle and lower esophagus for EoE and from the ascending colon for EGE. The tissues were fixed in 10% neutral-buffered formalin for 12–48 hours and routinely processed for formalin-fixed paraffin-embedded (FFPE) blocks and sections. For each sample, sets of three serial sections (3-μm thickness) of endoscopic biopsies were cut, and those without wrinkles, folds, tears, or detachment of paraffin were selected. Sections were stained with HE, MG, or DFS for eosinophil counting. The MG and DFS staining protocols are shown in Tables 2 and 3, respectively. To avoid inter-technical variability, all staining procedures in this study were performed by a single technician. Representative staining images of HE, MG and DFS-stained, are shown in Figure 1.

The histological sections were examined by three pathologists (A, C, D) and one cytotechnologist (B), with 36, 20, 13, and 15 years of experience in pathology practice, respectively. Pathologist A has been continuously involved in the diagnosis of EGIDs, and cytotechnologist B has occasionally diagnosed EGIDs together with pathologist A. Pathologists C and D were not familiar with the diagnosis of EGIDs. An outsider, who was not an examiner, selected the areas for eosinophil counting. The criteria for selecting areas were as follows: 1) presence of a characteristic landmark, e.g. crypt or blood vessel, that could be easily located, thus facilitating the establishment of the same precise observation field for all examiners; and 2) no biopsy artifacts, e.g. tissue crushing or excessive bleeding. A ZEISS microscope was used, with the characteristic gland or crypt located in the center of the field of view as a landmark, and all eosinophils present within the HPF (eye piece: 10× with a 23-mm field of view; objective lens: 40× with a numerical aperture of 0.95) were counted. Each examiner performed the EC twice in all specimens. The examiners were blinded to the background factors. The outsider analyzed the ECs by each examiner for each case.

### Digitalized image analyses

To characterize the color profiles of eosinophils and other cells and histological components, the JPEG-digitized photomicrographs were analyzed for brightness and red/green and yellow/blue saturation using Adobe PhotoShop Lightroom software (Adobe Systems Inc., San Jose, CA, USA). The CIE luminance-ab (L*a*b*) model^25^ was used for numerical quantification of eosinophil cytoplasmic granules. Three parameters were used to digitally characterize the color characteristics of eosinophils: brightness (L*), red/green (a*), and yellow/blue (b*) saturation. L* was scaled from 0 (black) to 100 (white). The a* and b* scales were defined as is, and scaled saturation values were defined as green (–100)/red (+100) and blue (–100)/yellow (+100). The white balance, which was set to L*=100, and a* and b* both at 0, was strictly adjusted before each photomicrograph was taken. For the three serial sections stained with HE, MG, and DFS, five spots in the captured images were selected for each slide. L*, a*, and b* values were measured for the eosinophil nuclei and granules, plasma cell cytoplasm, erythrocytes, and background stromal collagen fibers. The changes in L* values (ΔL) were determined using PhotoShop Lightroom, and the deviations from the origin of a* and b* (Δa and Δb) were calculated individually. The ΔL, Δa, and Δb values of eosinophil granules were compared with those of eosinophil nuclei, plasma cell cytoplasm, erythrocytes, and background stromal collagen fibers.

The total color difference (ΔE)^25^ was calculated as follows:

$$∆E=\sqrt{{{\left(∆L\right)}^{2}+\left(∆a\right)}^{2}+{\left(∆b\right)}^{2}}$$

The ΔE values of eosinophil granules were then compared with those of eosinophil nuclei, plasma cell cytoplasm, erythrocytes, and background stromal collagen fibers. The greater the numerical differences in color saturation (a* and b*) of these cells/components, the more clearly they could be distinguished by the human eye. The E-value is an index of color characteristics that integrates brightness and color saturation (red/green and yellow/blue), and roughly suggests that the higher the E-value of an object, the more easily distinguishable it is to the observer’s eye. Therefore, the greater the ΔE of two objects, the easier it is to distinguish them under a microscope.

### Statistical analyses

The color characteristics data of eosinophils, plasma cell cytoplasm, erythrocytes, and background stromal collagen fibers are expressed as the median and interquartile range. A linear mixed-effects model—using random effects at the participant level to account for repeated measures—was employed to assess associations among the time of measurement, type of stain (HE/MG/DFS), and examiner. Interactions among the time of measurement and type of stain were also included in the model. ECs among the examiners and types of stain were also compared using a linear mixed model and were adjusted for the time of measurement. The statistical significance of clinical data was analyzed using the Mann-Whitney U test with Bonferroni correction. Differences were considered statistically significant when the two-tailed *P* value was <0.05. SPSS version 27 (SPSS, Japan Inc., Tokyo, Japan) and R version 4.1.1 (The R Foundation for Statistical Computing, Vienna, Austria) were used for statistical calculations.

## Results

### Pathological examinations

**Figure 2:**
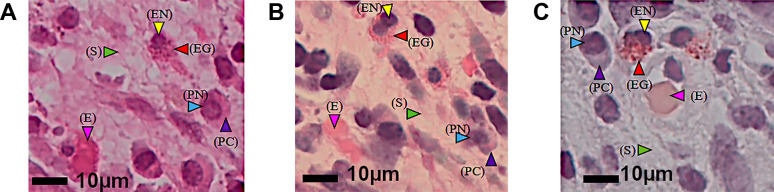
Standardization and digitization of microscopic brightness using JPEG format. At same time, white balance was performed for each image. Digital analyses of eosinophils and other cells under hematoxylin-eosin (HE, **A**), May-Grünwald Giemsa (MG, **B**), and direct fast scarlet (DFS, **C**) staining. The following characteristics were digitally analyzed for HE-, MG-, and DFS-stained slides: eosinophil nuclei (EN; yellow arrowhead), eosinophil granules (EG; red arrowhead), plasma cell cytoplasm (PC; purple arrowhead), plasma cell nuclei (PN; blue arrowhead), erythrocytes (E; pink arrowhead), and stromal collagen fibers (S; green arrowhead). For each stain, five points were measured with respect to the value of the Commission International de l’Eclairage luminance-a-b (L*a*b*) model. Scale bars=10 μm (**A**–**C**).

The eosinophil cytoplasm and granules were equally stained pink with HE and MG (Figures 1 and 2). With HE, the plasma cell cytoplasm and erythrocytes were also stained pink, but not as strongly as the eosinophil cytoplasm. With DFS, the eosinophil cytoplasm was selectively stained orange and easily distinguishable from other cells, including erythrocytes (Figure 1). The background stromal collagen fibers were weakly stained pink with HE and MG, but were not stained by DFS (Figure 2).

### Intra- and inter-observer variance in EC by staining method

The ECs for each stain by each examiner are shown in Table 4. We adopted a linear mixed-effect model to make a vertical comparison of the HE, MG, and DFS stains for ECs. The total ECs of all samples with all stains by all four examiners was not significantly different from the first and second rounds (*P*=0.294). Sections stained with MG or DFS had higher ECs than those stained with HE, with DFS delivering the highest ECs (β=35.151, 95% confidence interval [CI]: 27.220–43.083, *P*<0.001) (Table 5). The ECs of the four individual examiners differed, and were statistically significant (*P*<0.001) (Table 6). However, the closer the examiner was to expert level (A vs. B), the smaller the mean difference in the ECs from MG and DFS staining (HE: β=16.868, 95% CI: 12.340–21.397, *P*<0.001; MG: β=7.855, 95% CI: 4.112–11.599, *P*<0.001; DFS: β=9.145, 95% CI: 5.412–12.877, *P*<0.001) (Table 6). The most experienced examiner (A) reported the highest EC, while the least experienced examiner (D) reported the lowest EC. This finding suggested that inexperienced examiners may fail to count eosinophils correctly or mistake degranulated cytoplasmic granules of eosinophils for erythrocytes.

### Digitalized image analyses

There were no significant differences in the L* values of eosinophil granules with HE, MG, and DFS staining. There were no significant differences in the a* value of the eosinophil granules between MG and DFS, and there were no significant differences in the b* value of stromal collagen fibers between HE and MG. However, the other components showed significant differences in L*a*b* (Table 7).

The ΔE values for eosinophilic granules versus nuclei, plasma cell cytoplasm, erythrocytes, and background stromal collagen fibers were higher with MG and DFS staining than with HE staining. While MG staining produced the highest ΔE value for eosinophilic granules versus nuclei, DFS produced the highest ΔE values for all other cell/component comparisons. Additionally, there were significant differences in all items among the three stains (eosinophil granules versus eosinophil nuclei, plasma cell cytoplasm, erythrocytes, and stromal collagen fibers: *P*<0.001, <0.001, <0.001, <0.001) (Table 8).

## Discussion

In the present study, ECs were higher in EGIDs biopsy samples stained with MG and DFS than in those stained with HE, regardless of the individual examiner. Although there were differences in ECs between the experienced pathologists and the assistant cytotechnologist, these differences were reduced using MG and DFS staining. Therefore, MG and DFS staining may be useful for eosinophil counting.

EGE is considered a rare disease^10,28^; however, in Japan, the prevalence of EGE and other EGIDs is higher than that in other countries.^19^ The three types of EGE are classified on the basis of lesion histology: mucosa, muscularis, and subserosa.^29^ The clinical symptoms of EGE vary depending on the affected layer of the gastrointestinal tract. The most common manifestations of the mucosa are abdominal pain, nausea, and vomiting; those of the muscularis are gut wall thickness and bowel obstruction; and subserosal lesions manifest as eosinophilic ascites.^30^ The subserosal type occurs in only 10% of EGE cases.^31^ Endoscopy in EoE shows longitudinal furrows (48%), esophageal rings (44%), white exudates or plaques (27%), pallor or decreased vasculature (41%), and stricture (21%), but not specific for EoE.^32^ However, there are no characteristic endoscopic findings in EGE.^33^ CT scans of EoE show thickening of the esophageal wall in approximately 53% of cases.^19^ Diagnostic criteria for EGE include wall thickening and the presence of ascites,^17^ but the appearance rate of those is low.^31^ Zhang et al. (2011) reported that pathological findings are important for the diagnosis of EGIDs because there are no characteristic symptoms or findings in peripheral blood or imaging examinations.^34^ Manual counting of eosinophils under a microscope does not require special equipment and is easy to perform, but the accuracy of counting may depend on the competence and expertise of the individual examiner. We have shown here that the intra-observer variance in ECs was not significant (Table 5), whereas the inter-observer variance in ECs under HE, MG, and DFS staining was quite significant (Table 6). The ECs of the less experienced examiners (B, C, D) were lower than those of the well-trained examiner (A). These results indicate that, as the pathologist’s experience with eosinophil-related diseases increases, their ability to correctly identify eosinophils in tissue sections increases. Inter-observer variation in ECs among a group of pathologists examining slides from EoE patients was also reported by Vieira et al.^35^ Thus, the accuracy of eosinophil counting may be influenced by the amount of experience with EGIDs, allergic sinusitis/rhinitis, bronchial asthma, and other diseases in which eosinophils play an important role in the pathogenesis. We propose that years of experience as a general pathologist may be irrelevant for evaluating eosinophils and their cytoplasmic granules after degranulation; rather, the most important factor in achieving accurate ECs is familiarity with eosinophil-related diseases. We suspect that a future study among pathologists with experience in eosinophilic diseases might show lower inter-observer variance. All pathologists involved in the histological diagnosis of EGIDs should periodically review each other’s diagnostic skills to ensure that the uniform diagnostic standards of the pathology team are maintained.

Eosinophils are typically characterized by a double-lobed nucleus and bright pink cytoplasmic granules under HE staining. While the colors of HE-stained eosinophils, red blood cells, and collagen fibers of the tissue stroma may appear similar, their sizes and morphologic characteristics differ. Intact (non-granulated) eosinophils and their granules are approximately 17×12 μm and 500 to 1000 nm in size, respectively,^21,22^ whereas the average size of erythrocytes is ~8×2 μm. HE-stained, intact eosinophils can be clearly distinguished from erythrocytes by their condensed, double-lobed nuclei and cytoplasm filled with bright pink granules. However, if the eosinophil nuclei are not located within the paraffin section (3–4 μm thickness), the cytoplasm that does remain in the section may be confused with erythrocytes. Furthermore, if the eosinophil granules are degranulated but remain clustered rather than dispersed, they may be difficult to distinguish from erythrocytes. Additionally, collagen fibrils may look similar to erythrocytes/eosinophil granules when cross sectioned, and have a diameter of ~ 10 μm.^36^

Human color perception varies, and the power setting of the microscope light source allows for considerable adjustment to suit the observer’s preference. If the diagnosis of an EGIDs is to be made by several pathologists, the diagnostic criteria must be strictly standardized for accuracy. Talley et al. set the EC to ≥15/HPF for EoE and ≥20/HPF for EGE; however, in recent years, many authors have reported different EC cut-off values.^26,27^ We hypothesized that HE stain makes it difficult to count eosinophils, resulting in a difference in the cut-off values. To test this hypothesis, we digitally evaluated the color characteristics of eosinophil granules and other cellular components stained with HE, MG, and DFS using the color value of the CIE L*a*b* model.^25^ The larger the ΔE value, the larger the difference in color and thus ease of identification. We found that the ΔE values of eosinophilic granules versus nuclei, plasma cell cytoplasm, erythrocytes, and background stromal collagen fibers stained with MG and DFS were higher than those stained with HE. MG staining yielded the highest ΔE value for eosinophilic granules versus nuclei, while DFS yielded the highest ΔE values across all other comparisons. Comparing MG- and DFS-stained samples, differences in ΔE were observed in the plasma cell cytoplasm (MG vs. DFS=25.7 vs. 38.7, *P*<0.001), erythrocytes (MG vs. DFS=18.6 vs. 41.3, *P*<0.001), and stromal collagen fibers (MG vs. DFS=40.0 vs. 54.0, *P*<0.001) (Table 8). The ΔE of DFS was higher than that of MG in plasma cell cytoplasm, erythrocytes, and stromal collagen fibers; therefore, we reasoned that it was easier to locate the eosinophils in DFS-stained tissues. Additionally, we observed that eosinophil granules were stained pink by MG and orange by DFS, making DFS better for distinguishing eosinophils from other cells. We considered that eosinophils could be easily identified on the basis of eosinophil granule detection, and thus compared the ease of eosinophil counting of EGIDs sections using MG and DFS. To our knowledge, we are the first to report a quantitative evaluation of each staining method using CIE L*a*b*. While MG and DFS staining made it easier to visualize eosinophils compared with HE staining, they tended to result in higher ECs, which may make it difficult to distinguish EGIDs legions from other diseases, including reflux esophagitis/gastroesophageal reflux disease (GERD), or from healthy tissues. However, because the diagnostic criteria require the presence of symptoms,^10^ asymptomatic patients are not diagnosed with EoE and are described by the terms esophageal eosinophilia or esophageal eosinophilic infiltration, which indicate histologically eosinophil infiltration. Adachi et al. reported that esophageal eosinophil infiltration was observed in 0.4% (400/100,000) of asymptomatic patients who underwent esophageal biopsy.^37^ It is also known that eosinophils are present in GERD,^38^ and there has been much discussion about the differentiation between GERD and EoE. Although the current diagnostic criterion of ≥15 eos/HPF is used for EoE, our study found high ECs in sections stained with MG and DFS, thus this phenomenon is a topic for future consideration.

This study has several limitations. First, this was a single-center study with a relatively small sample size. Second, this retrospective study was inherently subject to selective bias. Third, because we did not perform immunohistochemical staining specific to eosinophils, we cannot be sure that the eosinophils identified by the most experienced examiner (A) were true eosinophils. However, we believe that examiner A recognized not only complete eosinophils, but also incomplete eosinophils. Fourth, staining of all sections was performed by a single technician; had multiple technicians been involved, the stainability of the sections may have differed. Fifth, although eosinophils are easier to find in acid-buffered formalin, it causes DNA fragmentation and nucleotide cross-linking, hampering genetic mutation analysis. Therefore, we used 10% neutral-buffered formalin, which is currently recommended for fixing pathological tissue specimens. Counting eosinophils in FFPE sections fixed in 10% neutral-buffered formalin and stained with HE requires experience; so we recommend adding MG or DFS staining for better visualization.

## Conclusions

Standardization of the diagnostic criteria for rare EGIDs is crucial, and will require the collaboration of several institutions and pathologists to obtain a sufficient number of cases to define, analyze, and establish detailed clinicopathological features. Our findings have highlighted potential problems in the current pathological approach to the diagnosis of EGIDs. We propose that utilizing MG and DFS staining for counting eosinophils in EGIDs sections offers greater ease and accuracy compared with HE staining.

## Figures and Tables

**Table1:** Background of the case patients in this study

characteristics	n (%)
EoE/EGE	10/28
Age	44.0 (34.8, 52.3)
Sex (male/female)	13/25
Other allergic diseases	
Food allergy	18 (47%)
Bronchial asthma	12 (32%)
Atopic dermatitis	4 (11%)
Peripheral blood test	
White Blood Cell (μL/ml)	6300 (5250, 7800)
Eosinophils (μL/ml)	58 (41, 67)
Basophiles (μL/ml)	224 (126, 327)
Neutrophils (μL/ml)	3588 (2622, 4298)
Lymphocytes (μL/ml)	1871 (1491, 2496)
Red Blood Cell (×10^3^ μL/ml)	476 (439, 503)
Haemoglobin (g/dL)	13.9 (12.8, 15.2)
Platelets (×10^3^ μL)	26.5 (22.1, 29.5)
C-reactive Protein (ng/mL)	0.07 (0.02, 0.14)
Symptoms (multiple choice allowed)	
Dysphagia	11 (29%)
Heartburn	3 (8%)
Abdominal pain	22 (58%)
Diarrhoea	20 (53%)
Melena	1 (3%)

Data expressed as the median with interquartile range. EoE: eosinophilic esophagitis; EGE: eosinophilic gastroenteritis.

**Table2:** May-Grünwald Giemsa staining procedure

1.	Deparaffinization (three consecutive baths of xylene, each five minutes)
2.	Rehydration (dipping the slides about a total of 20 times in three consecutive baths of 100%, then 70% ethanol)
3.	Rinse the slides with running tap water and then immerse in distilled water
4.	May-Grünwald stain^1)2)^ (40 minutes)
5.	Giemsa stain^1)3)^ (60 minutes)
6.	Differentiation (dipping the slide in 0.5% acetic acid^4)^ several times)
7.	Dehydration (several dips in four consecutive baths of 100% with isopropanol)
8.	Clearing with xylene (dipping the slides through four xylene baths) and mounting with cover slip

All staining steps are performed at room temperature. ^1)^ Dilute potassium sodium phosphate buffer (1/15 M, pH 6.4, Sigma-Aldrich Japan, Tokyo, Japan, cat. #40-0201) 10-fold with water. ^2)^ Mix 45 mL of May-Grünwald solution (Muto Pure Chemicals Co. Ltd., Tokyo, Japan, cat. #15054) with 90 mL of the diluted buffer and use for staining. ^3)^ Mix 2.4-mL of Giemsa solution (Merck Millipore, Tokyo, Japan, cat. #109204) with 120 mL of the diluted buffer and use for staining. ^4)^ Dilute glacial acetic acid with distilled water.

**Table3:** Direct fast scarlet (DFS) staining procedure

1.	Deparaffinization (three consecutive baths of xylene, each five minutes)
2.	Rehydration (dipping the slides about a total of 20 times in three consecutive baths of 100%, then 70% ethanol)
3.	Rinse the slides with running tap water
4.	Direct Fast Scarlet stain (DFS solution^1)^, 10 minutes)
5.	Rinse with running tap water for 5 minutes
6.	Nuclear stain (Mayer’s Haematoxylin^2)^, 5 minutes)
7.	Rinse with running tap water
8.	Dehydration with ethanol series (70%, 80%, 90%, and then 100% [three baths]) for a total of 5 minutes
9.	Clearing with xylene (dipping the slides through four xylene baths) and mounting with cover slip

All staining steps are performed at room temperature. ^1)^ Prepare DFS solution as follows: dissolve 0.1 g of DFS 4BS (Muto Pure Chemicals Co. Ltd., Tokyo, Japan, cat. #21421) in 50 mL of 50% ethanol; to this, add 0.4 g of sodium sulfate (anhydrate). Stir the solution for 10 min and filter before use. ^2)^ Muto Pure Chemicals Co. Ltd., Tokyo, Japan, cat. #30002.

**Table4:** Two rounds of eosinophil counts by each examiner using images of hematoxylin-eosin (HE)-, May-Grünwald Giemsa (MG)-, and direct fast scarlet (DFS)-stained tissue sections

Rater		Eosinophil counts							
		A		B		C		D	
		Median	IQR	Median	IQR	Median	IQR	Median	IQR
HE	One time	56	(25, 78)	38	(24, 57)	17	(8, 25)	5	(3, 8)
	Second time	55	(28, 77)	41	(25, 50)	20	(8, 26)	10	(7, 14)
MG	One time	58	(28, 80)	52	(26, 69)	33	(14, 55)	17	(9, 24)
	Second time	56	(30, 77)	52	(26, 64)	28	(15, 40)	16	(7, 24)
DFS	One time	84	(55, 109)	73	(52, 93)	51	(39, 68)	39	(25, 54)
	Second time	87	(58, 111)	77	(52, 92)	49	(30, 66)	35	(23, 41)

IQR: interquartile range.

**Table5:** Among the time of measurement and type of stain based on linear mixed-model analysis

		Coeficient (β)	95% LCL	95% UCL	*P* value
Repeat	Twice vs. one	1.908	–1.643	5.459	0.294
Type	MG vs. HE	13.053	5.121	20.984	0.001
	DFS vs. HE	35.151	27.220	43.083	<0.001

HE: hematoxylin-eosin stain; MG: May-Grünwald Giemsa stain; DFS: direct fast scarlet stain; LCL: lower confidence limit, UCL: upper confidence limit.

**Table6:** Discrepancy in eosinophil counts between two examiners using different staining methods

Staining	Compared Observers	Coeficient (β)	95% LCL	95% UCL	*P* value
HE	A to B	16.868	12.340	21.397	<0.001
	A to C	35.947	42.791	45.165	<0.001
	A to D	46.447	41.919	50.976	<0.001
	B to C	19.079	14.550	23.608	<0.001
	B to D	29.579	25.052	34.106	<0.001
	C to D	10.500	5.971	15.029	<0.001
MG	A to B	7.855	4.112	11.599	<0.001
	A to C	22.974	19.230	26.717	<0.001
	A to D	37.974	34.230	41.717	<0.001
	B to C	15.118	11.375	18.862	<0.001
	B to D	30.118	26.375	33.862	<0.001
	C to D	15.000	11.256	18.744	<0.001
DFS	A to B	9.145	5.412	12.877	<0.001
	A to C	33.303	29.570	37.035	<0.001
	A to D	47.513	43.781	51.246	<0.001
	B to C	24.158	20.425	27.890	<0.001
	B to D	38.368	34.636	42.101	<0.001
	C to D	14.211	10.478	17.943	<0.001

Data expressed as the median with interquartile range. HE: hematoxylin-eosin stain; MG: May-Grünwald Giemsa stain; DFS: direct fast scarlet stain; LCL: lower confidence limit, UCL: upper confidence limit.

**Table7:** Color characteristics of eosinophil granules and other cell types under staining with hematoxylin-eosin (HE), May-Grünwald Giemsa (MG), and direct fast scarlet (DFS)

Target		Color Values								
		HE		MG		DFS		HE vs. MG *P* value	HE vs. DFS *P* value	MG vs. DFS *P* value
		Median	IQR	Median	IQR	Median	IQR			
Eosinophil Granules	L*	54	(50, 57)	55	(51, 58)	54	(51, 58)	>0.999	>0.999	>0.999
	a*	58	(54, 61)	49	(44, 52)	48.5	(45, 53)	<0.001	<0.001	0.879
	b*	–4	(–6, –1)	–9	(–12, –7)	12	(9, 15)	<0.001	<0.001	<0.001
Eosinophil Nuclei	L*	39	(36, 44)	29	(25, 34)	41	(38, 46)	<0.001	0.012	<0.001
	a*	47	(40, 51)	29	(24, 33)	22	(18, 26)	<0.001	<0.001	<0.001
	b*	–15.5	(–19, –12)	–32	(–35, –29)	–12	(–15, –9)	<0.001	<0.001	<0.001
Plasma Cell Cytoplasm	L*	59	(56, 63)	43	(39, 50)	69	(64, 73)	<0.001	<0.001	<0.001
	a*	56	(53, 60)	37	(33, 40)	19	(17, 22)	<0.001	<0.001	<0.001
	b*	–7	(–9, –5)	–26	(–29, –23)	–8	(–10, –6)	<0.001	0.039	<0.001
Erythrocytes	L*	62	(59, 64)	70	(67, 73)	77	(73, 80)	<0.001	<0.001	<0.001
	a*	62	(56, 66)	42	(37, 46)	16	(9, 20)	<0.001	<0.001	<0.001
	b*	–0.5	(–4, 2)	–7	(–9, –5)	2	(–1, 5)	<0.001	0.000086	<0.001
Stromal collagen fibers	L*	75	(72, 78)	81	(76, 86)	85	(83, 87)	<0.001	<0.001	<0.001
	a*	35	(28, 41)	22	(14, 28)	6.5	(4, 9)	<0.001	<0.001	<0.001
	b*	–5	(–7, –4)	–5	(–8, –3)	–1	(–3, –1)	0.762	<0.001	<0.001

IQR: interquartile range; L*: measurement of the value of an object (L=0 black, L=100 white); a*: red-green measurement axis (positive is red, negative is green); b*: yellow-blue measurement axis (positive is yellow, negative is blue).

**Table8:** Differences in color values (ΔE) of eosinophil granules by staining method

Eosinophil Granules vs.	Color Value Differences (ΔE)			*P* values		
	HE	MG	DFS	HE vs. MG	HE vs. DFS	MG vs. DFS
Eosinophil Nuclei	42.5 (36.6, 46.8)	68.9 (62.8, 74.7)	46.8 (42.4, 53.2)	<0.001	<0.001	<0.001
Plasma Cell Cytoplasm	11.1 (8.1, 14.4)	25.7 (20.5, 30.8)	38.7 (33.6, 45.4)	<0.001	<0.001	<0.001
Erythrocytes	12.9 (9.6, 37.8)	18.6 (13.7, 25.1)	41.3 (35.6, 49.2)	<0.001	<0.001	<0.001
Stromal collagen fibers	32.1 (26.0, 37.8)	40.0 (30.7, 47.0)	54.0 (47.9, 58.0)	<0.001	<0.001	<0.001

Data expressed as the median with interquartile range. HE: hematoxylin-eosin stain; MG: May-Grünwald Giemsa stain; DFS: direct fast scarlet stain.

## Availability of Data and Material

All data generated or analyzed during this study are included in the article.
